# The Mutual Inhibition of FoxO1 and SREBP-1c Regulated the Progression of Hepatoblastoma by Regulating Fatty Acid Metabolism

**DOI:** 10.1155/2021/5754592

**Published:** 2021-09-08

**Authors:** Yu Hu, Hongyan Zai, Wei Jiang, Zhenglin Ou, Yuanbing Yao, Qin Zhu

**Affiliations:** Department of General Surgery, Xiangya Hospital, Central South University, Changsha, 410008, China

## Abstract

**Background:**

Hepatoblastoma (HB) is the most common liver malignancy in pediatrics, but the treatment for this disease is minimal. This study is aimed at exploring the effect of FoxO1 and SREBP-1c on HB and their mechanism.

**Methods:**

FoxO1, SREBP-1c, FASN, ACLY, ACC, and MAGL expressions in tissue samples were detected by RT-qPCR and WB. IHC was utilized to measure FASN content. Overexpression and knockdown of FoxO1 and sSREBP-1c were performed on Huh-6 cells. Cell proliferation, migration, and invasion were examined by CCK8, scratch, and transwell assay. ELISA was performed to test the ATP, FAO, NEFA, and Acetyl-CoA contents. ChIP was used to detect the interaction between SREBP-1c protein and the FoxO1 gene. In vivo tumorigenesis was conducted on mice. The morphology of tumor tissue sections was observed by HE staining.

**Results:**

FoxO1 expression was downregulated in HB tissue, while the expressions of SREBP-1c, FASN, ACLY, ACC, and MAGL were upregulated. In Huh-6 cells and mouse tumor tissues, FoxO1 knockdown resulted in increased cell proliferation, migration, and invasion and active fatty acid metabolism. On the contrary, after the knockdown of SREBP-1c, cell proliferation, migration, and invasion were weakened, and fatty acid metabolism was significantly reduced. SREBP-1c interacted with the promoter of the FoxO1 gene. When FoxO1 was knocked down, the tumor tissue was more closely packed. After the knockdown of the SREBP-1c gene, the structure of tumor cells was deformed.

**Conclusion:**

FoxO1 and SREBP-1c inhibited each other in HB, leading to the increase of intracellular fatty acid metabolism, and ultimately facilitated the development of HB.

## 1. Introduction

Hepatoblastoma (HB) is a pediatric tumor caused by hepatic progenitors or hepatoblasts. It is the most common liver malignant tumor in pediatrics. Its annual incidence is 1.5 cases per million, accounting for about 1% of all childhood cancers [1]. The primary treatment for HB is surgical resection, but about 60% of the tumors are unresectable at the onset, so the therapeutic effect is minimal [2]. Therefore, there is an urgent need to explore the pathogenesis of HB and develop new therapeutic targets to improve the clinical outcome of HB patients. One of the characteristics of cancer cells is reprogramming fatty acid metabolism [3]. Variation in lipid metabolisms, such as increased fatty acid uptake, de novo lipogenesis, is closely related to the generation of cancer cells [4]. The expression and activity of enzymes involved in lipid metabolism are significantly increased in many cancer cells, such as fatty acid synthase (FASN) and Acetyl-coenzyme A carboxylase (ACC) [5]. FASN plays a crucial role in lipid metabolism and has become an attractive target in clinical cancer treatment [6]. However, the mechanism of lipid metabolism in HB is still unclear.

The forkhead box-O1 (FoxO1) is a central regulator of metazoan physiology and plays a role in cell cycle, proliferation, apoptosis, autophagy, stress resistance, DNA repair, tumor inhibition, metabolism, and other cellular activities [7]. FoxO1 is tightly regulated by modifying its mRNA and protein, and its expression is regulated by nutritional signals in the environment [8]. Dysfunction of the FoxO1 pathway leads to various metabolic diseases, including diabetes, obesity, nonalcoholic fatty liver disease, and atherosclerosis [8]. FoxO1 is also thought to inhibit the development of osteosarcoma, but the mechanism of its inhibitory effect is not precise [9]. FoxO1 also plays a vital role in fat metabolism. It is reported that FoxO1 can slow down lipid deposition in the liver caused by stress response [10].

Sterol regulatory element-binding proteins are a class of transcription factors that regulate lipid homeostasis by controlling the synthesis of cholesterol, fatty acids, triglycerides, and phospholipids [11]. Among them, sterol regulatory element-binding protein-1c (SREBP-1c) is derived from the SREBP-1c gene on chromosome 17 and mainly regulates the synthesis of fatty acids and triglycerides [12]. It is an essential link between oncogenic signals and tumor metabolism [13]. Activation of SREBP-1c causes upregulation of FASN, enhances fatty acid metabolism, and theoretically promotes cancer development [14]. Geng et al. believed that SREBP-1c-driven lipid metabolism could be targeted to treat glioblastoma [15]. The regulatory pathway of FoxO1 and SREBP-1c in endometrial cancer was established [16]. FoxO1 inhibited insulin-induced SREBP-1c promoter activity in goat mammary epithelial cells and the transcription of SREBP-1c by the liver X receptor response element and SREBP response element on the SREBP-1c promoter [17]. However, it is still elusive whether FoxO1 and SREBP-1c play a role in regulating fatty acid metabolism in HB.

Derive from the above background, we wanted to explore the effect of FoxO1 and SREBP-1c on HB cells and study the mechanism of fatty acid metabolism in HB. Therefore, we collected clinical samples of HB and paracancerous tissues, purchased various HB cell lines, and conducted in vitro and in vivo experiments. This study contributes to our further understanding of the pathophysiology of HB and is expected to provide a new approach for the clinical treatment of HB patients.

## 2. Materials and Methods

### 2.1. Tissues and Cells

Clinical HB and paracancerous tissue samples were collected from Xiangya Hospital and divided into HB and control groups, with 5 samples in each group. The Human Research Ethics Committee of Xiangya Hospital approved this study (No. AF/SQ202104798). HB cells including HepG2 (bio-105877), HB611 (bio-73286), Huh-6 (bio-73060), and human normal liver cell WRL68 (bio-53604) were purchased from Biobw and cultured in DMEM medium (D5796, Sigma) containing 10% fetal bovine serum (#10099141, Gibco) at 37°C and 5% CO_2_. In order to investigate the effect of FoxO1, Huh-6 cells were randomly divided into 5 groups (the control, the oe-NC, the oe-FoxO1, the si-NC, and the si-FoxO1 groups). In the second majority of the study, Huh-6 cells were divided into 5 groups (control, si-NC, si-FoxO1, si-SREBP-1c, and si-FoxO1+si-SREBP-1c groups) to study the effect of SREBP-1c.

### 2.2. Vector Recombination and Cell Transfection

The si-FoxO1 and si-SREBP-1c vectors were obtained by integrating the shRNA sequence targeting human FoxO1 or SREBP-1c into a psi-LVRU6MP lentivirus vector (GeneCopoeia). The human FoxO1 cDNA sequence was inserted into the pCDH-CMV-MCS-EF1a vector (Epoch Life Science Inc) to get the oe-FoxO1 vector. The empty psi-LVRU6MP lentivirus vector was used as the si-FoxO1 vector. The empty pCDH-CMV-MCS-EF1a vector was the oe-FoxO1 vector. Then, the constructed vector was transfected into 293 T cells (HEK293T, Procell) to produce lentiviral solutions. These lentiviral solutions were transfected into Huh-6 cells with the assistance of 8 *μ*g/mL of Polybrene (#H9268, Sigma-Aldrich) [18]. Forty-eight hours after transfection [19], the cells were further examined.

### 2.3. Real-Time Quantitative Polymerase Chain Reaction (RT-qPCR)

The trizol method was used to extract total RNA from cells and tissues. cDNA was obtained by reverse transcription using an mRNA reverse transcription kit (#CW2569, Cowin Bio). Primer sequences of FoxO1, SREBP-1c, FASN, ATP-citric acid lyase (ACLY), ACC, monoacylglycerol lipase (MAGL), and *β*-actin were designed (Table 1). Shanghai Sangon Biotech synthesized the primers. Fluorescent dye was added to prepare the PCR reaction system. DNA amplification was performed by a fluorescent quantitative PCR apparatus (PIKOREAL96, Thermo). The amplification and fusion curves of each gene were obtained by real-time monitoring of fluorescence signals. *β*-Actin was used as an internal reference. The relative expression of genes was calculated using the 2^-*ΔΔ*CT^ method.

### 2.4. Western Blot (WB)

Total protein of cells and tissues was extracted with RIPA lysate (#P0013B, Beyotime). The mixture was bathed in water for 5 min after the protein supernatant was thoroughly mixed with the loading buffer. Then, the protein samples were isolated on gel and electrophoresis at a constant pressure of 75 V for 130 min. After electrophoresis, the target protein was transferred to the nitrocellulose membrane. The membranes were sealed and then incubated with primary antibodies FASN (1 : 2000, 10624-1-AP, Proteintech), ACLY (1 : 10000, 67166-1-Ig, Proteintech), ACC (1 : 4000, 21923-1-AP, Proteintech), MAGL (1 : 5000, ab124796, Abcam), and *β*-actin (1 : 5000, 60008-1-Ig, Proteintech) for 90 min. After incubation, the membranes were washed with PBST. The membranes and secondary antibody HRP goat anti-mouse IgG (SA00001-1, 1 : 5000, Proteintech) or HRP goat anti-rabbit IgG (SA00001-2, 1 : 6000, Proteintech) were then incubated for 90 min. Finally, the strips on the membranes were visualized using SuperECL Plus hypersensitive luminescence solution (K-12045-D50, Advansta). Grayscale values for all stripes were determined by Photoshop 2019. *β*-Actin was used as an internal parameter. The expression of the protein was expressed by the ratio of the grayscale value of the target protein to that of the reference protein.

### 2.5. Immunohistochemistry (IHC)

IHC detected the expression of FASN in tissues. Paraffin sections of HB and paracancerous tissues were made. After the sections were deparaffinized and rehydrated, they were heated in a microwave oven to repair the antigen. The endoenzymes were inactivated by adding 1% periodate acid to the sections. Then, sections and the anti-FASN antibody (10624-1-AP, 1 : 100, Proteintech) were incubated overnight at 4°C and then incubated with the second antibody for 30 min the next day. The sections were rinsed with PBS solution and incubated with DAB solution (ZSGB-BIO) for 5 min at room temperature. Sections were re-stained with hematoxylin for 5 min. The sections were treated with alcohol and xylene and sealed with neutral resin. Microscope (BA410T, MOTIC) was used to capture images, and the image analysis software was Image-Pro-Plus. The average IOD was calculated by the ratio of the cumulative optical density of the positive expression site to the sample area in view.

### 2.6. Cell Counting Kit-8 (CCK8) Assay

The cells were digested with trypsin and resuspended in a DMEM medium. Cells were seeded in a 5 × 10^3^ cells/well density in a 96-well plate of 100 *μ*L per well. The plates were placed in an incubator at 37°C and 5% CO_2_ for preculture. 10 *μ*L CCK8 solution (NU679, Dojindo) was added to each well. Cells were further incubated in the incubator for 4 h. Bio-Tek microplate analyzer (MB-530, HEALES) was used to measure the absorbance at 450 nm.

### 2.7. Scratch Assay

Trypsin was used to digest the cells in the logarithmic growth phase were digested into a single-cell suspension. Cells were seeded into a 6-well culture plate at a density of 5 × 10^5^ cells per well. The cells were cultured at 37°C in a 5% CO_2_ incubator for about 24 h until covered with six-well plates. A scratch was made with a pipette tip along the transverse line behind the six-well plates. The plates were washed three times with PBS to remove scratched cells. Then, serum-free DMEM medium was added. After being cultured for 0 h, 24 h, and 48 h, the cells were photographed under an inverted biological microscope (DSZ2000X, Cnmicro).

### 2.8. Transwell Assay

Transwell chamber (#3428, Corning) with a matrix gel (#354262, BD) was used to perform the transwell assay. The cells were digested into single-cell suspension with trypsin and resuspended in serum-free medium to 2 × 10^6^ cells/mL. 100 *μ*L cell suspension was inoculated in the up-compartment, and 500 *μ*L 10% DMEM/F12 medium (D8437, Sigma) was added in the low-compartment. It was incubated in an incubator at 37°C for 48 h. The cells in the up-compartment were rinsed with PBS solution. Cells were fixed with paraformaldehyde for 20 min, and the membrane was removed. The membrane was stained with 0.1% crystal violet for 5 min. Cells on the outer surface of the upper compartment were observed under an inverted biological microscope. After decolorization by acetic acid immersion, the cells' absorbance at 550 nm was measured with a microplate analyzer [20].

### 2.9. Fatty Acid Metabolism Detection

Nanjing Jiancheng Bioengineering Institute produced the ATP Assay Kit (#A095-1-1), Nonesterified Free Fatty Acids Assay Kit (#A042-2-1), and Triglyceride (TG) Assay Kit (#A110-1-1). Human Fatty Acid Oxidase (FAO) ELISA Kit (#JL48747) and Human Acetyl-Coenzyme A (Acetyl-CoA) ELISA Kit (#JL32777) were purchased from Jianglaibio (Shanghai, China). These kits were used to test adenosine triphosphate (ATP), FAO, TG, nonesterified fatty acid (NEFA), and Acetyl-CoA in cells. Each step strictly follows the instructions of the manual. Finally, the light signal at the specified wavelength was detected with a microplate analyzer [21].

### 2.10. Chromatin Immunoprecipitation (ChIP)

ChIP Kit (ab500, Abcam) was used to detect the direct interaction between SREBP-1c and FoxO1. After the cells were digested with trypsin, the cell suspension was incubated with formaldehyde and glycine to cross-link the target protein and the corresponding genomic DNA. Buffer D and protease inhibitors were added to the cell suspension. The mixture was ultrasonically crushed for 60 s and centrifuged. Agarose gel electrophoresis was performed to analyze the DNA fragment size. Then, immunoprecipitation was performed using agarose beads according to the instructions. Finally, the agarose beads were suspended with DNA purifying slurry to unlock the cross-linking and purify the DNA. Six pairs of primers were designed according to the FoxO1 gene's promoter (Table 2). RT-qPCR amplified DNA, and Fold Enrichment was calculated using the 2^-*ΔΔ*CT^ method.

### 2.11. In Vivo Tumorigenesis

Eight-week-old female nude mice (BALB/c, nu/nu) were purchased from the Animal Center of Central South University. Mice were randomly divided into 5 groups (*n* = 9): the control, the si-NC, the si-FoxO1, the si-SREBP-1c, and the si-FoxO1+si-SREBP-1c groups. Then, they were kept in captivity free of pathogens and given food and water at will. HepG2 cells were digested with trypsin and resuspended in a sterile salt solution. An equal number of HepG2 cells (2 × 10^5^) was subcutaneously injected into the lower abdomen of nude mice [21]. Tumor volume was measured weekly until the maximum volume was 1000 mm^3^. All mice were sacrificed with the manual cervical dislocation method. Tumor tissues were removed, measured, weighed, and further examined.

### 2.12. Hematoxylin and Eosin (HE) Staining

The tumor tissues of mice were made into paraffin sections. The sections were baked in the microwave oven at 60°C for 2 h. The sections were then deparaffinized in xylene and placed in 100%, 100%, 95%, 85%, and 75% ethanol for 5 min at each stage. The sections were soaked in distilled water and stained with hematoxylin for 5 min and eosin solution for 3 min. Then, the sections were dehydrated in gradient alcohol and soaked in xylene two times, each 10 min. Finally, they were sealed with neutral gum (Sigma), and photos were taken with an ordinary light microscope (BA210T, Motic).

### 2.13. Immunofluorescence (IF)

A microwave oven was used for baking the paraffin sections of mouse tumor tissues at 60°C for 2 h. The sections were deparaffinized and rehydrated by xylene and multiple concentration ethanol solutions. The slices were immersed in pH 6.0 citrate buffer (Wellbio). The citrate buffer was heated by the microwave oven for 24 min. After the buffer liquid was cooled, the sections were immersed in sodium borohydride solution for 30 min and Sudan Black solution for 5 min. Sections were sealed with 10% normal serum for 60 min. Sections were incubated overnight with primary antibody FASN (1 : 50, 10624-1-AP, Proteintech) at 4°C. On the second day, sections were incubated with the secondary antibody at 37°C for 90 min. Finally, sections were incubated with DAPI solution (Wellbio) at 37°C for 10 min and rinsed with PBS buffer. Sections were sealed with buffered glycerin and observed under a fluorescence microscope.

### 2.14. Statistical Analysis

Statistical analysis was performed using SPSS 20.0 (SPSS Inc, USA). Data were presented in the form of mean ± standard deviation (X̅±SD). All experiments were repeated at least three times. The Student *t*-test was used to analyze the differences between the two groups. Comparisons among multiple groups were conducted by one-way analysis of variance, followed by Tukey's post hoc test. *P* < 0.05 was considered statistically significant.

## 3. Results

### 3.1. FoxO1 Expression Was Downregulated while SREBP-1c and Fatty Acid Metabolism Genes Were Upregulated in HB

**Figure 1:**
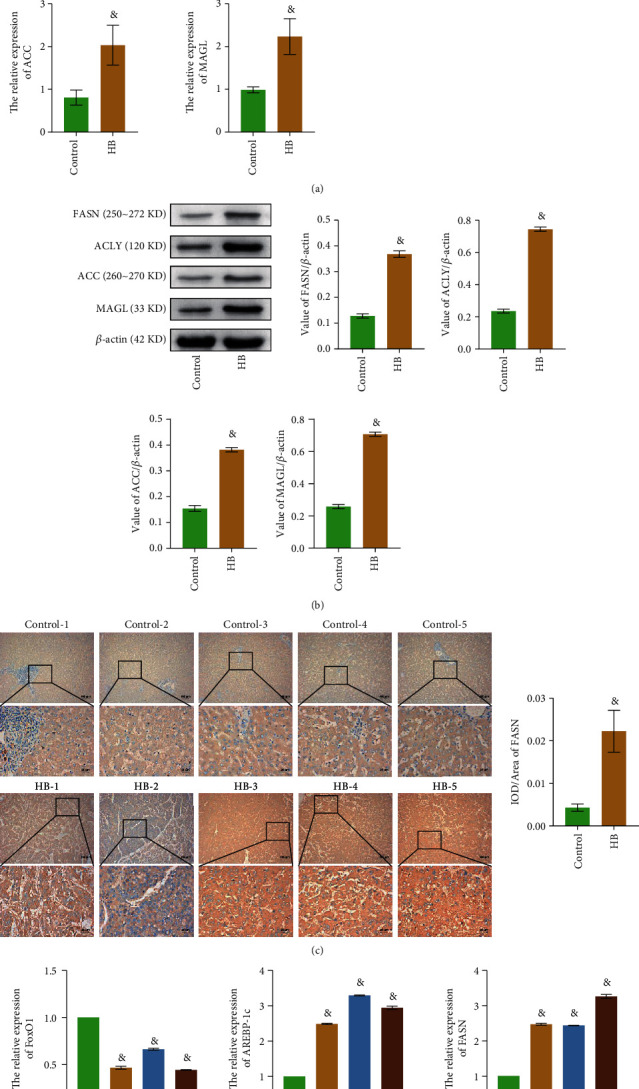
FoxO1 expression was downregulated while SREBP-1c and fatty acid metabolism genes were upregulated in HB. (a) The relative expression levels of FoxO1, SREBP-1c, FASN, ACLY, ACC, and MAGL were detected by RT-qPCR. (b) WB was used to measure the expressions of FASN, ACLY, ACC, and MAGL. (c) IHC evaluated the expression levels of FASN. (d) RT-qPCR was performed to examine the relative expressions of FoxO1, SREBP-1c, and FASN. The magnification is 100 or 400 times, and the corresponding scale bar is 100 *μ*m or 25 *μ*m; ^&^*P* < 0.05 vs. the control group in (a–c); ^&^*P* < 0.05 vs. the WRL68 group in (d). All experiments were performed 5 times.

In order to explore the changes of fatty acid metabolism in HB, RT-qPCR and WB detected the expressions of FoxO1, SREBP-1c, FASN, ACLY, ACC, and MAGL in clinical samples. As shown from Figure 1(a), compared with the control group, the FoxO1 expression in the HB group was decreased, and the SREBP-1c expression was significantly increased. At the level of RNA and protein, the expression levels of fatty acid metabolism-related indexes (FASN, ACLY, ACC, and MAGL) in the HB group were higher than those in the control group (Figures 1(a) and 1(b)). IHC results showed that FASN expression was upregulated in the HB group compared with the control group (Figure 1(c)). As shown in Figure 1(d), among the three kinds of HB, the difference in gene expression between Huh-6 cells and normal liver cells (WRL68) was the most significant, so Huh-6 cells were selected for subsequent experiments.

### 3.2. FoxO1 Deficiency Led to Upregulation of SREBP-1c Expression and Enhanced Proliferation, Migration, Invasion of HB

**Figure 2:**
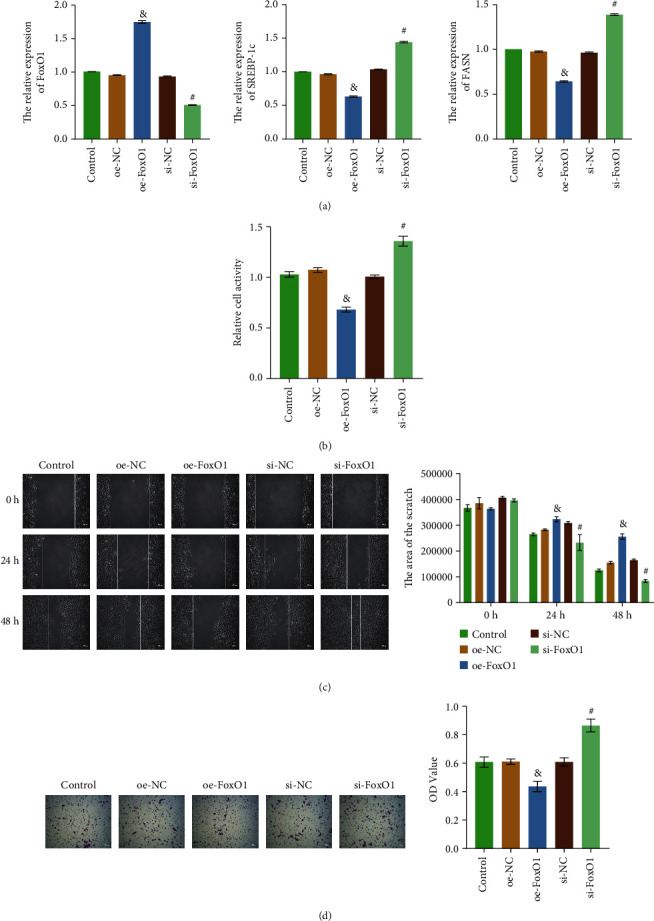
FoxO1 deficiency led to upregulation of SREBP-1c expression and enhanced proliferation, migration, invasion of HB. (a) RT-qPCR detected the relative expression levels of FoxO1, SREBP-1c, and FASN. (b) CCK8 assay was used to detect cell proliferation. (c) Cell migration was evaluated by scratch assay. (d) Transwell assay was performed to examine cell invasion. The magnification is 100 times, scale bar = 100 *μ*m; ^&^*P* < 0.05 vs. the oe-NC group; ^#^*P* < 0.05 vs. the si-NC group. All experiments were performed 3 times.

In order to determine the effect of FoxO1 on SREBP-1 expression and cell function in HB, Huh-6 cells with FoxO1 overexpression or knockdown were constructed. RT-qPCR detected the expression levels of FoxO1, SREBP-1c, and FASN, and the results suggested that FoxO1 overexpression or knockdown cells were completed (Figure 2(a)). Compared with the oe-NC group, the expression of SREBP-1c and FASN in the oe-FoxO1 group decreased. Compared with the si-NC group, the expression levels of SREBP-1c and FASN in the si-FoxO1 group were increased (Figure 2(a)). As shown in Figures 2(b)–2(d), cell proliferation, migration, and invasion abilities in the oe-FoxO1 group were weaker than those in the oe-NC group. Compared with the si-NC group, cell proliferation, migration, and invasion abilities of the si-FoxO1 group were enhanced. In other words, FoxO1 could inhibit the expression of SREBP-1c and FASN and reduce the proliferation, migration, and invasion abilities of Huh-6 cells.

### 3.3. FoxO1 Deficiency Enhanced Fatty Acid Metabolism in HB

**Figure 3:**
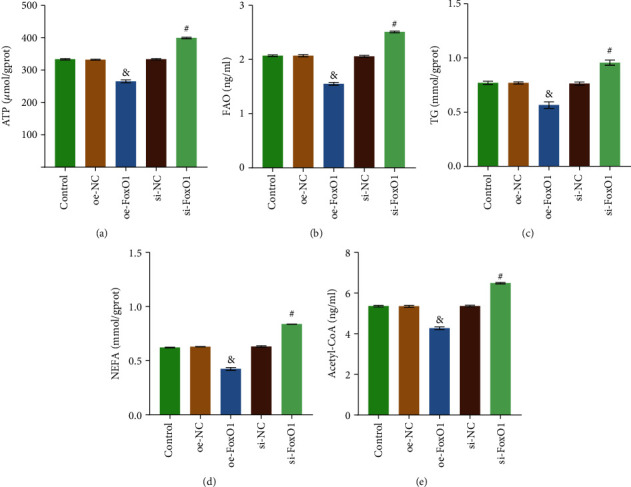
FoxO1 deficiency enhanced fatty acid metabolism in HB. (a) ATP Assay Kit was used to detect the concentration of total cellular ATP. (b) Human FAO ELISA Kit was adopted to examine FAO. (c) TG Assay Kit detected TG. (d) NEFA was examined with Nonesterified Free Fatty Acids Assay Kit. (e) Cellular Acetyl-CoA level was evaluated with Human Acetyl-CoA ELISA Kit. ^&^*P* < 0.05 vs. the oe-NC group; ^#^*P* < 0.05 vs. the si-NC group. All experiments were performed 3 times.

Intracellular fatty acid metabolism in both overexpression and deletion of FoxO1 was examined to clarify the effect of FoxO1 on fat metabolism in HB. As shown in Figures 3(a)–3(e), compared with the oe-NC group, ATP, FAO, TG, NEFA, and Acetyl-CoA contents in the oe-FoxO1 group decreased. In the meantime, compared with the si-NC group, the contents of ATP, FAO, TG, NEFA, and Acetyl-CoA in the si-FoxO1 group increased (Figures 3(a)–3(e)). These results suggested that FoxO1 could inhibit the uptake and production of ATP, TG, NEFA, and Acetyl-CoA. It also inhibits the FAO. In other words, FoxO1 inhibited fatty acid metabolism (including anabolism and catabolism) in Huh-6 cells.

### 3.4. FoxO1 and SREBP-1c Inhibited Each Other and Regulated Huh-6 Cell Functions

**Figure 4:**
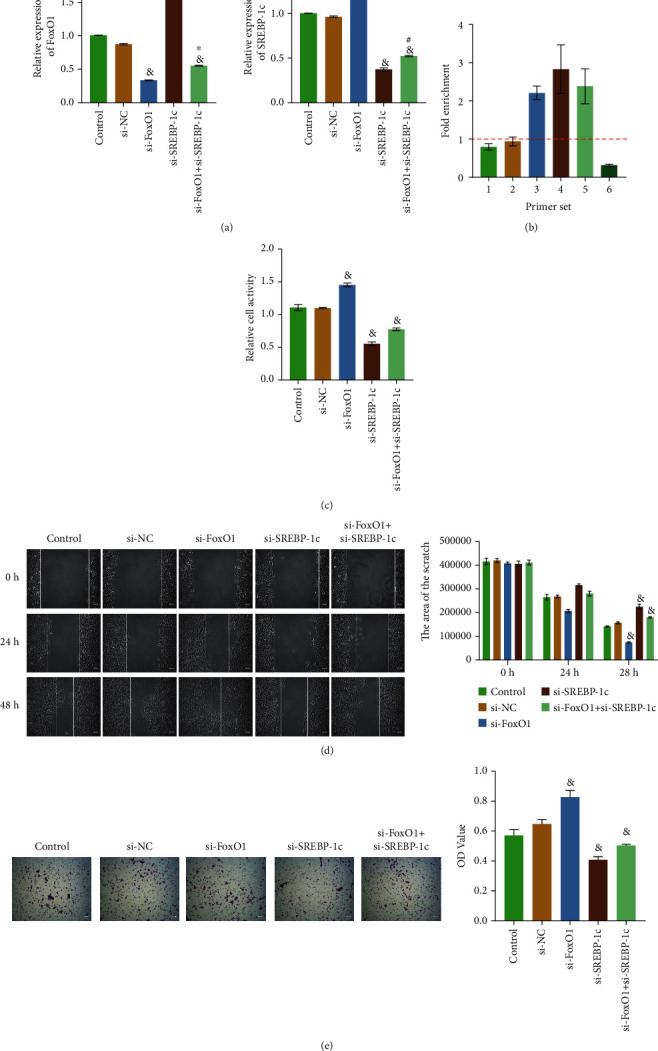
FoxO1 and SREBP-1c inhibited each other and regulated Huh-6 cell functions. (a) RT-qPCR detected the relative expression levels of FoxO1 and SREBP-1c. (b) ChIP was performed to detect the direct interaction between the FoxO1 gene and SREBP-1c protein. (c) CCK8 assay was used to detect cell proliferation. (d) Cell migration was evaluated by scratch assay. (e) Transwell assay was performed to examine cell invasion. The magnification is 100 times, scale bar = 100 *μ*m; ^&^*P* < 0.05 vs. the si-NC group, ^∗^*P* < 0.05 vs. the si-FoxO1 group, and ^#^*P* < 0.05 vs. the si-SREBP-1c group. All experiments were performed 3 times.

It has been previously confirmed that FoxO1 has a regulatory effect on SREBP-1c and HB. To further explore how FoxO1 and SREBP-1c work together, Huh-6 cells that FoxO1 and SREBP-1c knockdown at the same time or SREBP-1c knockdown alone were constructed.  Figure 4(a) showed that SREBP-1c could inhibit FoxO1 expression (fold change ≈ 1.65), and FoxO1 also inhibited SREBP-1c expression (fold change ≈ 1.39). We could see that SREBP-1c had a more substantial inhibitory effect on FoxO1 than FoxO1 on SREBP-1c (fold change ≈ 1.65 > 1.39). Then, ChIP was performed to study whether there was a direct interaction between SREBP-1c and FoxO1 gene. As shown in Figure 4(b), the Fold Enrichment of primers 3, 4, and 5 was greater than 1, indicating that the enrichment capacity of nonspecific adsorption of antibodies was less than the specific action of antibodies. It showed that SREBP-1c directly interacted with sites 3, 4, and 5 of the FoxO1 gene's promoter. Cell function results exhibited that compared with the si-NC group, the cells' proliferation, migration, and invasion abilities in the si-SREBP-1c group were reduced. In contrast, those in the si-FoxO1 group were enhanced (Figures 4(c)–4(e)). When FoxO1 and SREBP-1c were knocked down simultaneously, cells' proliferation, migration, and invasion abilities were reduced. These results suggested that FoxO1 inhibited cell proliferation, migration, and invasion, while SREBP-1c had the opposite effect. Meanwhile, FoxO1 and SREBP-1c inhibit each other, and their net effect in Huh-6 cells was to promote cell proliferation, migration, and invasion.

### 3.5. Coordinated Regulation of FoxO1 and SREBP-1c Regulated Fatty Acid Metabolism in Huh-6 Cells

**Figure 5:**
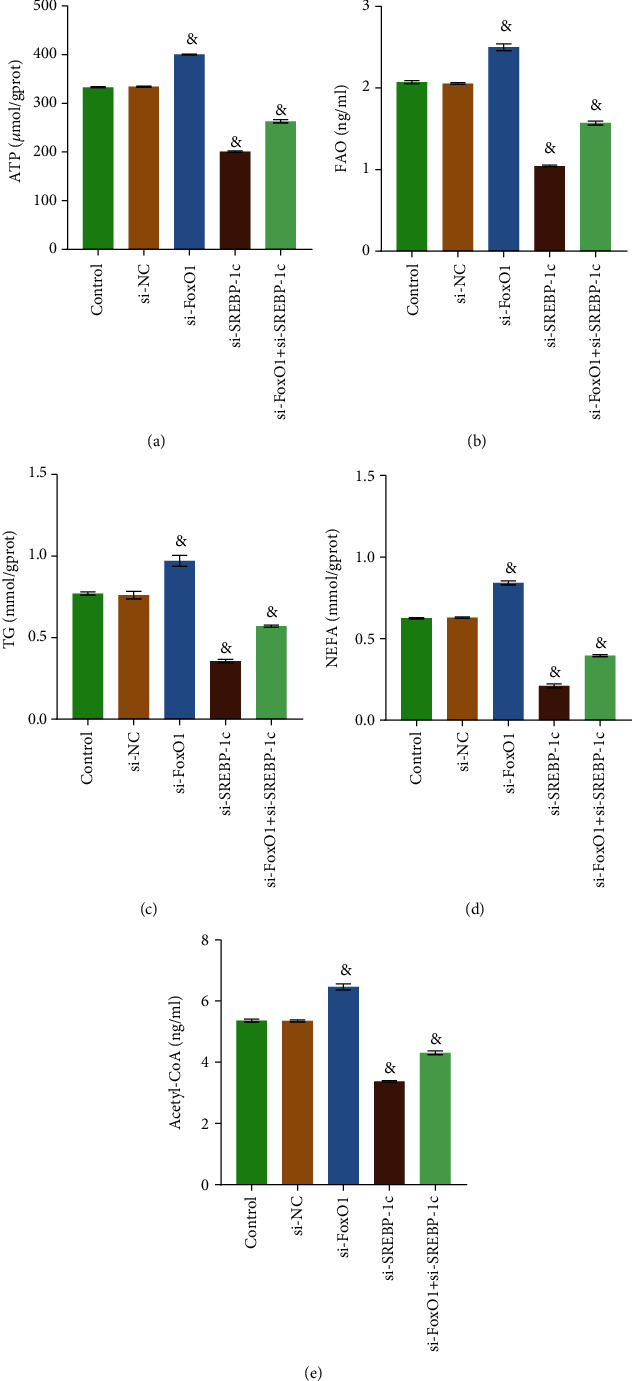
Coordinated regulation of FoxO1 and SREBP-1c regulated fatty acid in Huh-6 cells. (a) ATP Assay Kit was used to detect the concentration of total cellular ATP. (b) Human FAO ELISA Kit was adopted to examine FAO. (c) TG Assay Kit detected TG. (d) NEFA was examined with Nonesterified Free Fatty Acids Assay Kit. (e) Cellular Acetyl-CoA level was evaluated with Human Acetyl-CoA ELISA Kit. ^&^*P* < 0.05 vs. the si-NC group. All experiments were performed 3 times.

We have demonstrated that FoxO1 has a regulatory effect on SREBP-1c and fatty acid metabolism in HB cells. To study how FoxO1 and SREBP-1c play roles in regulating fatty acid metabolism, we constructed Huh-6 cells that knocked down both FoxO1 and SREBP-1c or knocked down SREBP-1c alone for the detection of fatty acid metabolism-related indicators. As shown in Figures 5(a)–5(e), compared with the si-NC group, the contents of ATP, FAO, TG, NEFA, and Acetyl-CoA decreased in the si-FoxO1+si-SREBP-1c group and the si-SREBP-1c group, while that increased in the si-FoxO1 group. These results indicated that the net effect of FoxO1 and SREBP-1c was to promote fatty acid metabolism in HB cells.

### 3.6. Coordinated Regulation of FoxO1 and SREBP-1c Facilitated the Progression of HB by Regulating Fatty Acid Metabolism In Vivo

**Figure 6:**
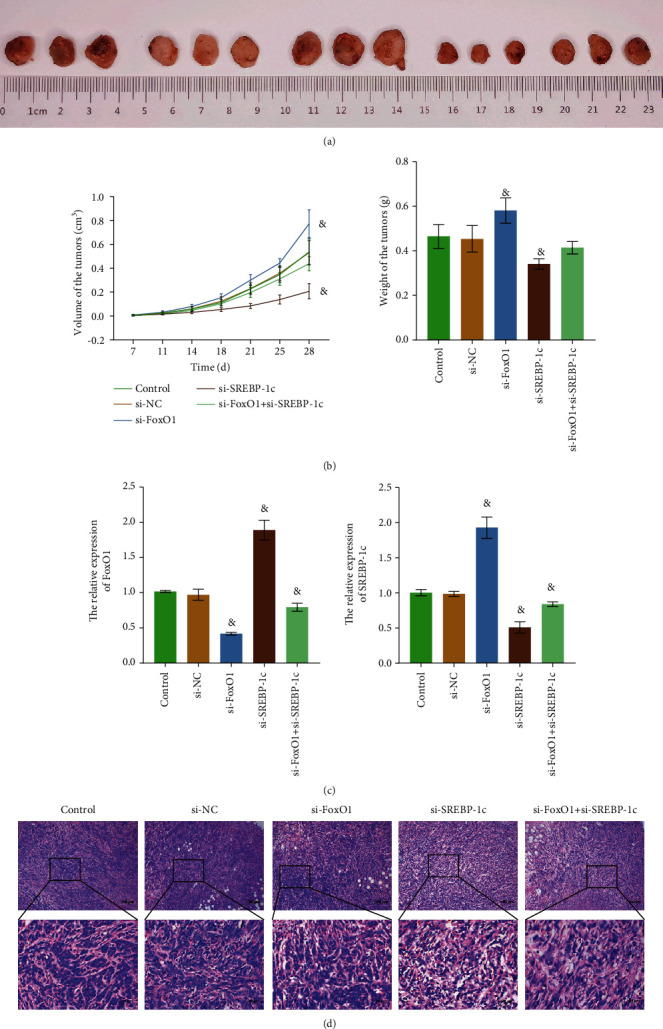
Coordinated regulation of FoxO1 and SREBP-1c facilitated the progression of HB in vivo. (a) Photograph of subcutaneous neoplasia in nude mice. (b) Volume and weight of tumor tissues. (c) The relative expression levels of FoxO1 and SREBP-1c were detected by RT-qPCR. (d) HE staining was performed to observe tumor tissues. The magnification is 100 or 400 times, and the corresponding scale bar is 100 *μ*m or 25 *μ*m; ^&^*P* < 0.05 vs. the si-NC group. All experiments were performed 3 times.

**Figure 7:**
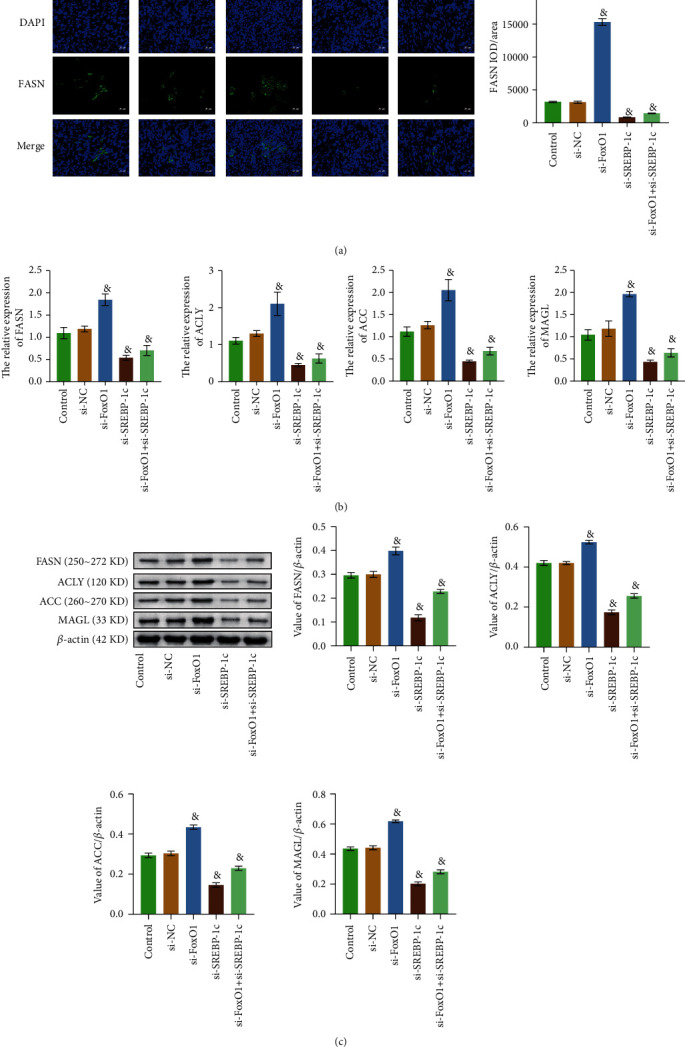
Coordinated regulation of FoxO1 and SREBP-1c regulated fatty acid metabolism in vivo. (a) The concentration of FASN was detected by IF. (b) The relative expression levels of FASN, ACLY, ACC, and MAGL were evaluated by RT-qPCR. (c) WB was used to measure the expressions of FASN, ACLY, ACC, and MAGL. The magnification is 400 times, scale bar = 25 *μ*m; ^&^*P* < 0.05 vs. the si-NC group. All experiments were performed 3 times.

Previous experiments were all conducted in vitro. Subcutaneous tumor-forming models of nude mice were constructed, and tumor tissues were collected to verify whether the results of the in vivo experiments were consistent with those in vitro.  Figure 6(a) showed that the subcutaneous tumorigenesis model of nude mice was successfully constructed. As shown from Figure 6(b), compared with the si-NC group, the tumor volume and weight of the si-FoxO1 group increased significantly, while those of the si-SREBP-1c group decreased. RT-qPCR results demonstrated that knockdown of FoxO1 and SREBP-1c genes was successfully realized in tumor tissues (Figure 6(c)). As shown in Figure 6(d), compared with the si-NC group, the tumor tissue structure and morphology were regular and tightly arranged in the si-FoxO1 group. In si-SREBP-1c and si-FoxO1+si-SREBP-1c groups, the structure of tumor tissues was damaged. Compared with the si-NC group, the expression levels of FASN, ACLY, ACC, and MAGL in the si-FoxO1 group were increased in the si-SREBP-1c and si-FoxO1+si-SREBP-1c groups were decreased (Figures 7(a)–7(c)). Tumor tissue detection results showed that FoxO1 and SREBP-1c inhibited each other, and the net effect of FoxO1 and SREBP-1c facilitated the progression of HB by regulating fatty acid metabolism in vivo.

## 4. Discussion

Metabolic reprogramming in cancer cells has been recognized as one of the basic features of cancer [22]. In gastric cancer, SREBP-1c is activated and fatty acid synthesis is significantly increased [23]. Neoadipogenesis and fatty acid *β*-oxidation are very active in hepatocellular carcinoma [24]. Our analysis of clinical samples indicates that the expression of FoxO1 was downregulated in HB tissues, and the expression of genes of SREBP-1c and key enzymes in fatty acid metabolism were significantly upregulated. Naturally, the activation of SREBP-1c and fatty acid metabolism is preliminarily considered a significant characteristic of HB. FoxO1 degradation promotes cell proliferation in colon cancer [25]. Interestingly, FoxO1 overexpression in esophageal cancer promotes tumor development by increasing macrophage infiltration [26]. After cervical cancer, the proliferation, migration, and invasion abilities are significantly enhanced [27]. In summary, FoxO1 has various functions and regulates the progression of multiple types of cancer through numerous pathways. In this study, we find that the knockdown of FoxO1 promotes the development of HB. SREBP-1c is a crucial protein in fatty acid metabolism [28]. It activates the transcription of FASN, a major fat-generating gene, which promotes the growth of bladder cancer [29]. In our study, when SREBP-1c was knocked down, the proliferation, migration, invasion, and division abilities of HB cells were reduced, and the fatty acid metabolism level was also significantly decreased.

In the study of diabetic cardiomyopathy, Ying et al. found that FoxO1 has a regulatory effect on fatty acid metabolism [30]. The ATP level of cancer cells is much higher than that of normal differentiated cells to meet the energy needs of growth and proliferation [31]. Usually, differentiated cells rely primarily on mitochondrial oxidative phosphorylation to produce ATP, a process that uses three main biofuels: glucose, glutamine, and fatty acids, while the proliferation of cancer cells depends on the FAO [32]. FAO is significantly enhanced in human glioblastoma, and inhibition of FAO leads to decreased intracellular ATP level and viability [33]. We detected ATP and FAO in HB. Results indicated that the knockdown of FoxO1 could significantly increase FAO in HB and keep ATP at a high level. In other words, FoxO1 deficiency promotes catabolism of fatty acids in HB. Acetyl-CoA is a precursor of fatty acid and cholesterol synthesis [34]. ACLY-dependent Acetyl-CoA production plays a crucial role in the early stages of pancreatic neoplasia [35]. NEFAs are organic compounds with variable linear chain lengths of 6-32 carbons and hydrophilic heads containing a carboxylic acid, promoting colon, lung, skin, and breast cancer [36]. Lipids stored in lung neutrophils are transported to metastatic tumor cells through the micropinocytosis-lysosome pathway, which enhances the survival and proliferation of tumor cells [37]. Our results showed that FoxO1 knockdown significantly increased Acetyl-CoA, NEFA, and TG levels in HB. In other words, FoxO1 deficiency can promote the anabolism of fatty acids in HB. Epigallocatechin gallate suppresses hepatic cholesterol synthesis by targeting SREBP-2 through SIRT1/FoxO1 signaling pathway [38]. In our study, FoxO1 knockdown accelerated HB cells' energy production, enhanced fatty acid metabolism, and ultimately promoted the development of HB cells. This result is consistent with previous studies.

Many studies have shown that FoxO1 could affect fatty acid metabolism by regulating the expression of SREBP-1c. For example, knockdown of FoxO1 significantly increased SREBP-1c and FASN in hepatitis C virus-infected cells [39]. Deng et al. found that FoxO1 could disrupt the assembly of key components of the SREBP-1c promoter transcription complex and inhibit the activity of the SREBP-1c promoter, thereby inhibiting the expression of SREBP-1c [40]. However, the mechanism by which FoxO1 plays a role in HB remained unclear. Through knockdown and overexpression of FoxO1, our study proved that FoxO1 could also inhibit the expression of SREBP-1c and inhibit fatty acid metabolism in HB. Interestingly, we found that SREBP-1c also had an inhibitory effect on FoxO1 (Figure 4(a)). There were few reports about the inhibitory effect of SREBP-1c on FoxO1, so we performed a ChIP experiment to prove the direct interaction between SREBP-1c and FoxO1. Some studies also supported that SREBP-1c could inhibit the expression of FoxO1 through indirect action. For example, Sajan et al. found that atypical protein kinase C activated by SREBP-1c inactivated FoxO1 via WD40/PROF (a scaffold protein)-associated Akt in diabetes [41]. Therefore, it was essential to determine their net effect on HB growth. We knocked down FoxO1 and SREBP-1c simultaneously in Huh-6 cells and found that fatty acid metabolism of Huh-6 cells was inhibited, and cell function and tumor-forming ability were weakened. In other words, coordinated regulation of FoxO1 and SREBP-1c could facilitate the progression of HB by regulating fatty acid metabolism.

In this study, we found mutual inhibition of FoxO1 and SREBP-1c in HB. In addition, SREBP-1c could bind to the promoter of FoxO1 to regulate its transcription. However, the mechanism of how FoxO1 inhibits SREBP-1c expression remains unclear. As mentioned earlier, SPREP-1c also inhibits FoxO1 expression through indirect regulation. In HB, it is still unclear whether direct or indirect regulation plays a dominant role. We were unable to carry out a detailed study on this part because of insufficient experimental funds. In the future, we will conduct a series of molecular biology experiments to refine our research.

## 5. Conclusion

FoxO1 could slow down the progress of HB by inhibiting the fatty acid metabolism while SREBP-1c promotes it. FoxO1 and SREBP-1c have an inhibitory effect on each other. Coordinated regulation of FoxO1 and SREBP-1c facilitated the progression of HB by regulating fatty acid metabolism in vivo and vitro. These findings provided a theoretical basis for a better understanding of the mechanism of fatty acid metabolism in HB. They helped to develop new targets for the clinical treatment of HB.

## Figures and Tables

**Table 1:** RT-qPCR primer sequences.

Gene	Sequences (5′-3′)	Product length (bp)
*FoxO1*	F: ACTTCATCTCATTCTCCCTTCTGC	199
	R: GCACAACTTACAGCTGGTTTTCAA	

*ACLY*	F: CCTCAGCCATCCAGAATCGG	194
	R: CTTCAGCCAGGACTTGACCC	

*SREBP-1c*	F: GCTCCCTAGGAAGGGCCGTA	240
	R: CACTCTTAGTTTTCCTTCCGTTT	

*FASN*	F: CCTGGCTGCCTACTACATCG	102
	R: CACATTTCAAAGGCCACGCA	

*ACC*	F: CTCTTGGCCTTTTCCCGGTC	228
	R: GTTATCCCCAAACCCAGGCA	

*MAGL*	F: TCCAGCATGCCAGAGGAAAG	142
	R: TGGGACACAAAGATGAGGGC	

*β-Actin*	F: ACATCCGTAAAGACCTCTATGCC	224
	R: AGCACAGCCTGGATAGCAAC	

**Table 2:** FoxO1 primer sequences.

Primer	Sequences (5′-3′)	Product length (bp)
1	F: CAGAACCCCATGGCTAAGGTC	153
	R: ATCTAATCCTGGCTCATTCCT	

2	F: ACACTGAGGGTCCATCCCA	165
	R: AGTTTTCACACTGAACTGTGCAT	

3	F: TGTTAGACTTTGTAGCCGGACAG	125
	R: TGGCCGATTCACAGATCAAGA	

4	F: ACACTGGAAGACCTTTGCCTT	108
	R: GAACAGCCCTCCACCTACCTT	

5	F: GGATTGGGGTACAAGTCCAC	168
	R: GGTTTCCTGATGTATTACCCAC	

6	F: CCCCATATTTCCACGAACTCCA	159
	R: AGGACAAATAACAAGCGACCTTC	

## Data Availability

The data used to support the findings of this study are available from the corresponding authors upon request.

## Abbreviations

HB: Hepatoblastoma
FASN: Fatty acid synthase
ACC: Acetyl-coenzyme A carboxylase
FoxO1: Forkhead box-O1
SREBP-1c: Sterol regulatory element-binding protein-1c
ACLY: ATP-citric acid lyase
MAGL: Monoacylglycerol lipase
TG: Triglyceride
FAO: Fatty acid oxidase
Acetyl-CoA: Acetyl-coenzyme A
NEFA: Nonesterified fatty acid.
